# The Ecology of *Acidobacteria*: Moving beyond Genes and Genomes

**DOI:** 10.3389/fmicb.2016.00744

**Published:** 2016-05-31

**Authors:** Anna M. Kielak, Cristine C. Barreto, George A. Kowalchuk, Johannes A. van Veen, Eiko E. Kuramae

**Affiliations:** ^1^Department of Microbial Ecology, The Netherlands Institute of Ecology – Koninklijke Nederlandse Akademie van WetenschappenWageningen, Netherlands; ^2^Graduate Program in Genomic Sciences and Biotechnology, Universidade Católica de BrasíliaBrasília, Brazil; ^3^Ecology and Biodiversity Group, University of UtrechtUtrecht, Netherlands

**Keywords:** *Acidobacteria*, carbohydrate metabolism, transporters, nitrogen metabolism, EPS, soil factors

## Abstract

The phylum *Acidobacteria* is one of the most widespread and abundant on the planet, yet remarkably our knowledge of the role of these diverse organisms in the functioning of terrestrial ecosystems remains surprisingly rudimentary. This blatant knowledge gap stems to a large degree from the difficulties associated with the cultivation of these bacteria by classical means. Given the phylogenetic breadth of the *Acidobacteria*, which is similar to the metabolically diverse *Proteobacteria*, it is clear that detailed and functional descriptions of acidobacterial assemblages are necessary. Fortunately, recent advances are providing a glimpse into the ecology of members of the phylum *Acidobacteria*. These include novel cultivation and enrichment strategies, genomic characterization and analyses of metagenomic DNA from environmental samples. Here, we couple the data from these complementary approaches for a better understanding of their role in the environment, thereby providing some initial insights into the ecology of this important phylum. All cultured acidobacterial type species are heterotrophic, and members of subdivisions 1, 3, and 4 appear to be more versatile in carbohydrate utilization. Genomic and metagenomic data predict a number of ecologically relevant capabilities for some acidobacteria, including the ability to: use of nitrite as N source, respond to soil macro-, micro nutrients and soil acidity, express multiple active transporters, degrade gellan gum and produce exopolysaccharide (EPS). Although these predicted properties allude to a competitive life style in soil, only very few of these prediction shave been confirmed via physiological studies. The increased availability of genomic and physiological information, coupled to distribution data in field surveys and experiments, should direct future progress in unraveling the ecology of this important but still enigmatic phylum.

## Introduction

Although the *Acidobacteria* were only recognized as a phylum relatively recently, their abundance across a range of ecosystems, especially soils, has demanded research into their ecology. 16S rRNA gene-based approaches as well as environmental shotgun metagenomic analyses have revealed that the *Acidobacteria* represent a highly diverse phylum resident to a wide range of habitats around the globe (Chow et al., 2002; Kuske et al., 2002; Gremion et al., 2003; Quaiser et al., 2003; Fierer et al., 2005; Stafford et al., 2005; Janssen, 2006; Sanguin et al., 2006; de Carcer et al., 2007; Kim et al., 2007; Singh et al., 2007; DeAngelis et al., 2009; Jesus et al., 2009; Kielak et al., 2009; Navarrete et al., 2010, 2013b; Zhang et al., 2014). However, despite their high abundance and diversity, we still have relatively little information regarding the actual activities and ecology of members of this phylum, a shortcoming that can be attributed to a large extent to the difficulties in cultivating the majority of acidobacteria and their poor coverage in bacterial culture collections (Bryant et al., 2007; Lee et al., 2008; da Rocha et al., 2009; Eichorst et al., 2011; Navarrete et al., 2013b). However, environmental surveys have provided insight into some the environmental factors that may drive acidobacteria dynamics, such as pH and nutrients (Fierer et al., 2007; Jones et al., 2009; Lauber et al., 2009; Navarrete et al., 2013b).

In 2009, the first sequenced genomes of acidobacteria strains became available, providing preliminary genetic insights into the potential physiology and environment functions of several members of this phylum (Ward et al., 2009). In these first genomic studies, five aspects of physiological received particular attention: (i) carbon usage, (ii) nitrogen assimilation, (iii) metabolism of iron, (iv) antimicrobials, and (v) abundance of transporters. Besides genome sequencing of cultivated isolates, addition information regarding genomic properties of acidobacteria has been derived from metagenomics studies (Liles et al., 2003; Quaiser et al., 2003, 2008; Riaz et al., 2008; Jones et al., 2009; Kielak et al., 2010; Parsley et al., 2011; Faoro et al., 2012; Navarrete et al., 2013b; Mendes et al., 2014).

In this review, we couple the complementary data coming from physiological, genomic and metagenomics studies to seek a better understanding of the role of *Acidobacteria* in the environment, thereby providing some initial insights into the ecology of this important phylum. We aim to not only give a more complete picture of the current knowledge of *Acidobacteria*, but also seek to provide a solid base for future experiments geared toward gaining a better understanding of the ecological roles played by members of this phylum.

## History and General Information on the Phylum *Acidobacteria*

The introduction of molecular biological strategies into microbial ecology over the past decades has yielded a new perspective on the breadth and vastness of microbial diversity. The phylum of the *Acidobacteria* is one of the bacterial lineages that has profited most from the cultivation-independent interrogation of environmental samples. Indeed, in the past two decades, this phylum has grown from being virtually unknown to being recognized as one of the most abundant and diverse on Earth. This phylum is particularly abundant in soil habitats that can represent up to 52% from the total bacterial community (Dunbar et al., 2002; Sait et al., 2002) and averaging approximately 20% of the microbial community across diverse soil environments (Janssen, 2006).

Although 16S rRNA gene sequences related to the *Acidobacteria* were obtained as early as 1993 (Stackebrandt et al., 1993) it was only in 1997 that they were associated with sequences belonging to cultured members of the current *Acidobacteria* phylum. Based on phylogenetic analysis of 16S rRNA gene sequences, the *Acidobacteria* phylum raised from the originally described four to six subdivisions (Kuske et al., 1997; Ludwig et al., 1997; Barns et al., 1999, 2007) to over eight subdivisions in 1998 (Hugenholtz et al., 1998) and in 2005 this number increased to 11 (Zimmermann et al., 2005) deeply branching and strongly supported subdivisions. Currently there are 26 accepted subdivisions (Barns et al., 2007) in the Ribosomal Database Project. The first recognized strain and species of the phylum *Acidobacteria* was *Acidobacterium capsulatum* obtained from an acid mine drainage in Japan (Kishimoto and Tano, 1987; Kishimoto et al., 1991; Abed et al., 2002). Although the second isolate belonging to this phylum was *Holophaga foetida* first described in 1994, it was not initially recognized as related to *Acidobacteria capsulatum.* Instead, it was thought to belong to the phylum *Proteobacteria* (Liesack et al., 1994). A few years later, a closely related bacterium named *Geothrix fermentans* was isolated (Coates et al., 1999) and subsequently another closely related bacterium *Acanthopleuribacter pedis*, the first acidobacteria obtained from a marine sample, was described (Fukunaga et al., 2008). Since these isolates were very distantly related to *A. capsulatum*, it was proposed that it should be included in a new class named *Holophagae. Acidobacteriia* and *Holophagae* are the only classes currently included in the most recent edition of the Bergey’s Manual (Thrash and Coates, 2014).

Currently *Acidobacteria* phylum has 26 subdivisions based on the extremely broad diversity of acidobacterial populations found in uranium-contaminated soils (Barns et al., 2007). Newly characterized acidobacteria from subdivision 1 may challenge this taxonomy in the near future, since of 16S rRNA gene analysis has consistently shown that the genera *Acidobacteria,‘Acidipila*’ (Okamura et al., 2011), *Telmatobacter* (Pankratov et al., 2012), and *Acidicapsa* (Kulichevskaya et al., 2012) form a group that is distinct from the genera *Granulicella, Terriglobus, Bryocella*, and *Edaphobacter* (Eichorst et al., 2007; Koch et al., 2008; Pankratov and Dedysh, 2010; Männistö et al., 2011, 2012; Rawat et al., 2012a,b; Baik et al., 2013; Whang et al., 2014).

**FIGURE 1:**
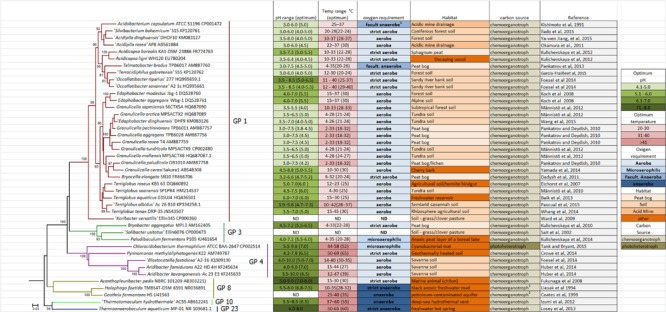
**Dendrogram showing the general characteristics of *Acidobacteria* type species, only.** The dendrogram was obtained using nearly-full 16S rRNA gene sequences obtained and aligned using the Ribosomal Database Project. Dendrogram were obtained with MEGA7 (Tamura et al., 2011) using the Neighbor-joining method (Saitou and Nei, 1987); bootstrapping values were based on 1000 repetitions and are shown next to the branches (Felsenstein, 1985). There were a total of 1343 positions in the final dataset. 1. *Acidobacterium capsulatum* was originally described as an aerobic bacteria, but later it was demonstrated a weak anaerobic growth by fermentation (Pankratov et al., 2012). 2. *H. foetida* is homoacetogen and *G. fermentans* is fermentative. ND, not determined. Quote marks (‘) indicate that this species is deposited in a culture collection, but its name does not retain its validity and standing in nomenclature.

The vast majority of isolates cultivated to date are affiliated with acidobacteria subdivision 1 (Class *Acidobacteriia*). They are all heterotrophic, most species are aerobic or microaerophilic and some species (*Telmatobacter bradus, Acidobacterium capsulatum*) are facultative anaerobic bacteria (Pankratov et al., 2012). Members of subdivisions 3, 4, 8 (currently Class *Holophagae*), 10, and 23 are heterotrophic as well. *Thermotomaculum* (subdivision 10) and *Thermoanaerobaculum* (subdivision 23) are thermophilic anaerobic bacteria (Izumi et al., 2012; Losey et al., 2013). *Chloracidobacterium thermophilum* is photoheterotrophic (Bryant et al., 2007; Tank and Bryant, 2015) and *Pyrinomonas methylaliphatogenes* can consume H_2_ (Greening et al., 2015), both from subdivision 4. Subdivision 8 contains one aerobic (*Acanthopleuribacter*) and two strictly anaerobic isolates (*Holophaga* and *Geothrix*). There are reports of acidobacteria isolates belonging to subdivisions 2 and 6, but they still do not have valid taxonomic names (Sait et al., 2002; George et al., 2011; Parsley et al., 2011). Subdivisions 1 and 3 of the phylum *Acidobacteria* together with thermophilic *Thermoanaerobacter* species are capable of biosynthesizing total fatty acids lipid (Damste et al., 2011). Currently, there a total of 40 species belonging to 22 genera: eleven genera of subdivision 1, two of subdivision 3; four of subdivision 4, three of subdivision 8, one of subdivision 10, and one of subdivision 23 (**Figure 1**). In addition, there are the genome sequences of *Koribacter* and *Solibacter*, but there is little information on their physiology.

## The Impact of New Isolation Methods

Changes in the traditional methods for culturing bacteria from soils have significantly improved the isolation of *Acidobacteria* strains in recent years. These new strategies involve the use of relatively low concentration of nutrients, non-traditional sources of carbon or complex polysaccharides (Janssen et al., 2002; Sait et al., 2002; Pankratov et al., 2008; Eichorst et al., 2011), longer periods of incubation, the use of gellan gum as solidifying agent, (Janssen et al., 2002; Sait et al., 2002), non-standard CO_2_ atmospheric conditions of incubation, addition of quorum-signaling molecules, catalase or cations (Stevenson et al., 2004; George et al., 2011; Navarrete et al., 2013a), amendments of inhibitors for undesired organisms, and amendment of environmental extracts in growth media (Foesel et al., 2013).

It is suggested that raising the CO_2_ concentration may not only better mimic the CO_2_ concentrations typically found in soils, but may also decrease medium pH, thereby benefiting certain members of the acidobacteria, especially moderate acidophilic strains belonging to subdivision 1 (Sait et al., 2006). This combination of strategies seems to enrich not only for *Acidobacteria* but for many other groups of slow-growing bacteria. The association of a molecular technique such as the high-throughput plate-wash PCR (Stevenson et al., 2004) or colony-PCR (de Castro et al., 2013), both using phylum-specific 16S rRNA gene primers has improved the screening and identification of colonies belonging to the *Acidobacteria* subgroup 1. Once *Acidobacteria* isolation under low nutrient conditions is achieved, strains can often be transferred to richer media (e.g., TSA and R2A) for more convenient propagation (de Castro et al., 2013). Despite the importance of these recent advances in cultivation methods, further improvements are clearly needed since only eight of a total of 26 subdivisions are known to have representatives in culture. A large number of *Acidobacteria* isolates have been recovered from the Australian soil Ellin bank (Janssen et al., 2002; Sait et al., 2002; Davis et al., 2005). These studies were important during the development of new strategies for culturing soil bacteria, and two of these isolates, ‘*Koribacter versatilis’* Ellin345 (CP000360) and ‘*Solibacter usitatus*’ Ellin6076 (CP000473), were used in the first *Acidobacteria* genome investigations (Ward et al., 2009). However, many of these bacteria have not yet been fully characterized and still do not possess valid taxonomical names. Further, micro-cultivation strategies combined with single-cell sequencing should provide access to new acidobacterial genomes, and in turn this genomic information may help to inform future isolation efforts as cultivation is still required for most physiological characterizations.

## The Impact of *Acidobacteria* Genomes and Links to Physiological Studies

The first comparative genome analysis between of *A. capsulatum* and two bacteria originated from the Ellin collection, ‘*S. usitatus*’ Ellin6076 (subdivision 1), and ‘*K. versatilis*’ Ellin345 (subdivision 3) provided numerous insights into the physiology of members of the *Acidobacteria* (Ward et al., 2009; Challacombe et al., 2011). Since then, the number of acidobacterial genomes being sequenced remains rather limited. Currently, there are 10 published genomes of *Acidobacteria* available: five subdivision 1 (Ward et al., 2009; Rawat et al., 2012a,b, 2013, 2014), one subdivision 3 (Ward et al., 2009), two subdivision 4 (Costas et al., 2012; Lee et al., 2015), and one subdivision 8 – class *Holophagae* (Anderson et al., 2012); 1 subdivision 23 (Stamps et al., 2014). Below, we summarize some of the major findings revealed via the currently available acidobacterial genome sequences linked to physiological studies.

## Carbohydrate Metabolism

**FIGURE 2:**
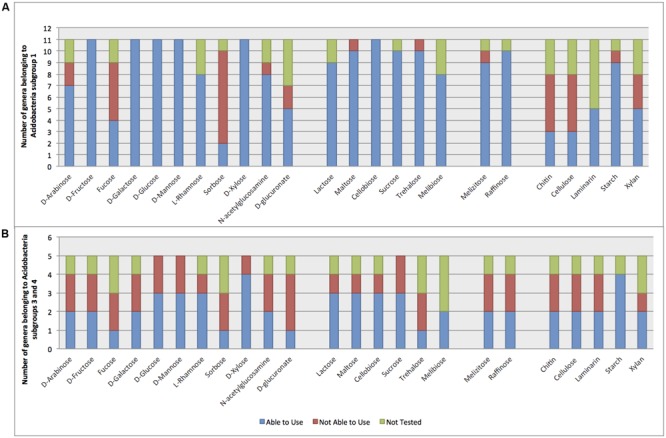
**Usage of carbon sources by *Acidobacteria* in culture-based experiments with type strain species.** A positive score was recorded if at least one species within a genus is able to use a respective sugar. **(A)** *Acidobacteria* subdivision 1. **(B)** *Acidobacteria* subdivisions 3 and 4. Usage of carbon source obtained from original references that described each of the type species, in order of publication: Kishimoto et al., 1991; Liesack et al., 1994; Coates et al., 1999; Eichorst et al., 2007; Fukunaga et al., 2008; Koch et al., 2008; Kulichevskaya et al., 2010, 2012, 2014; Pankratov and Dedysh, 2010; Männistö et al., 2011, 2012; Okamura et al., 2011; Dedysh et al., 2012; Izumi et al., 2012; Pankratov et al., 2012; Baik et al., 2013; Foesel et al., 2013, 2016; Losey et al., 2013; Crowe et al., 2014; Huber et al., 2014; Whang et al., 2014; Yamada et al., 2014; Pascual et al., 2015; Tank and Bryant, 2015; Garcia-Fraile et al., 2016; Jiang et al., 2016; Llado et al., 2016).

Among all the physiological aspects revealed by genomics, carbohydrate metabolism has been studied most widely, which is not surprising considering that carbon usage is one of the physiological requirements for the description of new species in taxonomic studies. At least one species of each of the eight recognized genera of subdivision 1 is able to use D-glucose, D-xylose, and lactose as carbon sources (**Figure 2**). The ability to use glucose and xylose makes sense given the fact that cellulose or xylan are often the major carbon sources in the culture media most typically used for the isolation of *Acidobacteria*. In addition, these bacteria were able to use most of the tested oligosaccharides, although maltose and cellobiose were not able to support growth of *Edaphobacter* species. Interestingly, the majority of subdivision 1 species were unable to use fucose or sorbose, carbohydrates that are only minor components of plant cell walls and rather scarce in soil (Li et al., 2013).

Although several acidobacterial genomes have been shown to contain genes encoding for the degradation of different polysaccharides (**Figure 2**), experimental data on the use of polysaccharides generally do not support genomic predictions. At least 50% of the genera have members able to use starch, lamminarin, and xylan. In contrast, chitin usage has not yet been demonstrated for any member of *Acidobacteria* subdivision 1. Similarly, cellulose was another substrate predicted to be degraded by *Acidobacteria* genome annotation. However, only *Telmatobacter bradus* (subdivision 1) has been demonstrated to be able to use crystalline cellulose (Pankratov et al., 2012) and *Edaphobacter cerasi* (Yamada et al., 2014) is able to grow on CM-cellulose. *Terracidiphilus gabretensis* produces extracellular enzymes implicated in the degradation of plant-derived biopolymers what was confirmed by genome analysis by the presence of enzymatic machinery required for organic matter decomposition (Garcia-Fraile et al., 2016). In contrast to *Acidobacteria* from subdivision 1, members of subdivision 4 are able to use chitin as a carbon source (Foesel et al., 2013; Huber et al., 2014) (**Figure 2**). Most of the *Acidobacteria* subdivisions 1, 3, or 4 examined to date is unable to use carboxymethyl cellulose, but there is evidence that *Aridibacter kavangonensis* (subdivision 4) is able to utilize micro-crystalline cellulose (Huber et al., 2014). Although it is still premature to draw general conclusions related to the degradation of these abundant polysaccharides by *Acidobacteria* in nature, xylan degradation has been broadly demonstrated, which may play a role in plant cell wall degradation (Pankratov et al., 2008; Eichorst et al., 2011).

The discrepancies between genome predictions and observed activities may stem from our ability to provide cultivation conditions that lead to the expression of the target activities. Alternatively, current automatic genome annotation pipelines may not successfully differentiate genes involved for instance in chitin and cellulose degradation from genes involved in the degradation of other glycosyl hydrolases, such as xylan. Systematic studies on the degradation of cellulose by *Acidobacteria* grown on different culture conditions may help to test the hypothesis of gene regulation by sugars present in the media, for example. On the other hand, it has been reported that in bacteria many genes involved in cellulose degradation may be involved in the infection of plant cells or in the synthesis of bacterial cellulose (Koeck et al., 2014).

**FIGURE 3:**
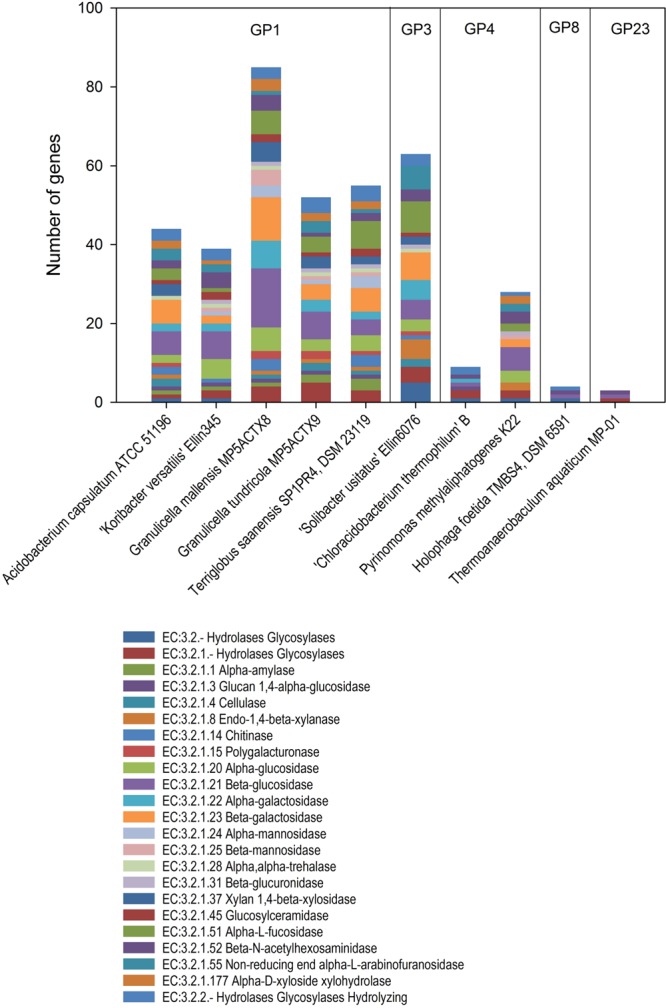
**Enzymes encoding genes of polysaccharide degradation (EC.3.2-) in different *Acidobacteria* subdivisions (GP) genomes.** The comparisons were done using IMG: the integrated microbial genomes database and comparative analysis system.

Enzymatic activities observed in *Acidobacteria* have usually been detected using commercial kits with chromogenic substrates. Members of subdivision 1 possess a broader range of enzymes related to sugar usage than those from other subdivisions (**Figure 3**; Supplementary Table S1). Galactosidases are enzymes involved in the hydrolysis of galactose-containing sugars, while beta galactosidades are involved in the degradation of lactose. Since all genera of *Acidobacteria* subdivision 1 are able to use lactose, it is not surprising to find this enzyme included in their enzymatic profile. Glucosidases are involved in the degradation of polysaccharides, especially cellulose and starch. Although starch is used by most *Acidobacteria* (**Figure 2**), cellulose degradation has not yet been unequivocally demonstrated for most *Acidobacteria*, as explained above.

Interestingly, β-glucosidase has successfully been purified and characterized from *A. capsulatum* (Abed et al., 2002). This enzyme was induced by the presence of cellobiose in the medium, and it was active on *p*-nitrophenyl-*p*-D-glucopyranoside (100%), cellobiose (39%), and β-gentiobiose (33%). In a subsequent study, the gene *xyn*A for a endo-β-1,4-xylanase from *A. capsulatum* (Koch et al., 2008) was cloned and expressed in *Escherichia coli* (Dedysh et al., 2012). This enzyme was demonstrated to be active on xylan and cellobiose (100% of relative activity), but has low activity on CM-cellulose (5.4%) and no activity on filter paper of Avicel.

Janssen et al. (2002) were the pioneers to use gellan gum as solidifying agent in culture medium used for *Acidobacteria* isolation. In contrast to agar, which is obtained from seaweed, gellan gum is a substrate that is produced (and degraded) by soil bacteria. At least two species of *Acidobacteria* have demonstrated ability to use gellan gum: *Telmatobacter bradus* and *Bryocella elongata*. One known pathway for the degradation of gellan-gum involves the action of gellan lyases, α-L-rhamnosidases, unsaturated glucuronyl hydrolases, and β-D-glucosidases (Baik et al., 2013). Members of *Acidobacteria* subdivision 1 were reported to be able to use the monosaccharides rhamnose and glucose. Also, at least one β-D-glucosidase was characterized from *A. capsulatum*, which may be involved in gellan gum degradation. However, the *in silico* comparison of 10 available genomes, offered no evidence of gellan lyase (EC 4.2.2.25) genes for gellan lyase usage. Moreover, Naumoff and Dedysh (2012) reported that the presence of α-rhamnosidases is not a phylogenetically determined trait, but that this function was obtained by lateral gene transfer from *Bacteroidetes* and in the case of *Holophaga foetida* from a fungus. The investigation of the enzymatic pathway for gellan gum degradation may merit further investigation, since this is a bacterial polysaccharide. Therefore, in addition to the possible metabolism of polysaccharides derived from plants, the usage of gellan gum suggests an interaction with other soil bacteria (Dedysh et al., 2012).

## Nitrogen Metabolism

Nitrite reduction was observed in all three genomes reported in 2009, and nitrate reduction in two of the initially analyzed genomes (Ward et al., 2009). Nitrate reduction has been investigated in almost all members of subdivision 1, with the exception of *Acidobacterium* and *Acidicapsa.* Among all *Granulicella* species, *G. mallensis* was reported to perform nitrate reduction (Männistö et al., 2012). *A. rosea* and *B. elongata* are also able to reduce nitrate to nitrite. Among other subdivisions, only *Geothrix fermentans* subdivision 8 was shown to be able to reduce nitrate. This organism is an iron reducer that can use nitrate as an alternative electron acceptor. All of these *Acidobacteria* were able to use yeast extract that, in addition to ammonium, may be a preferred nitrogen source (Coates et al., 1999). The presence of the *nirA* gene, which encodes nitrate reductase, also appears to be limited to subdivision 1, suggesting that members of this subdivision may reduce nitrate to nitrite by the assimilatory pathway, which is further reduced to ammonia and assimilated into glutamate. Nevertheless, the direct uptake of ammonium seems likely as all genomes described to date appear to contain genes for the ammonia transporter channel (Amt) family (TC 1.A.11). Nitric oxide reductase genes (*norB* but not *norC*) were identified in ‘*Koribacter versatilis*,’ *‘Solibacter usitatus*’ and *Geothrix fermentans* genomes. Genes encoding dinitrogenase, a heterotetramer of the proteins NifD and NifK (genes *nifD* and *nifK*, respectively) and dinitrogenase reductase, a homodimer of the protein NifH (gene *nifH*), were found only in the genome of *H. foetida*. Ammonia monooxygenase (*amo*) and nitrous-oxide reductase (*nosZ*) genes were not found in any of the available genomes. Contrary to the previous report (Ward et al., 2009), homologs of nitrate reductases *narB* and *narG* were not found in available genome sequences via *in silico* genome comparison, and physiological testing would therefore be required to suggest this function in *Acidobacteria*. In summary, there is no clear evidence for the involvement of *Acidobacteria* in key *N*-cycle processes such as nitrogen fixation, nitrification, or denitrification.

## Exopolysaccharides

Exopolysaccharide (EPS) production has frequently been reported in cultured *Acidobacteria* species (Eichorst et al., 2007; Pankratov and Dedysh, 2010; Whang et al., 2014). Initial genomic analyses revealed that at least one *Acidobacteria* encodes genes involved in the EPS biosynthesis, specifically for the production of bacterial cellulose, genes involved in the cellulose synthesis are encoded on genomes belonging to subdivision 1 (exception *‘K. versatilis*’ Ellin345). However, it is not known if this is a general characteristic of the phylum *Acidobacteria*. It has been suggested that EPS-producing bacteria may be able to survive for long periods in soil due to the protection provided by their EPS. The dominance of *Acidobacteria* in acidic environments and its resistance to pollutants like uranium (Ellis et al., 2003; Gremion et al., 2003; Barns et al., 2007), petroleum compounds (Abed et al., 2002), linear alkylbenzene sulfonate (Sanchez-Peinado et al., 2010) and *p*-nitrophenol (Paul et al., 2006) may therefore be related to the ability to produce large amounts of EPS.

The functions of EPS in soil are numerous. It may be involved in the formation of the soil matrix, may serve as a water and nutrition trap, and may be involved in bacterial adhesion that can facilitate soil aggregate formation (Eichorst et al., 2007, 2011; Pankratov and Dedysh, 2010). Based upon the presence of cellulose synthesis genes and a large number of novel high-molecular-weight proteins with excretion pathway motifs, it has been postulated that many *Acidobacteria* have the potential to form biofilms, resist desiccation and facilitate soil aggregate formation. However, to date, there are still no physiological studies demonstrating actual acidobacterial EPS production or demonstration of its ecological role.

## Transporters

**FIGURE 4:**
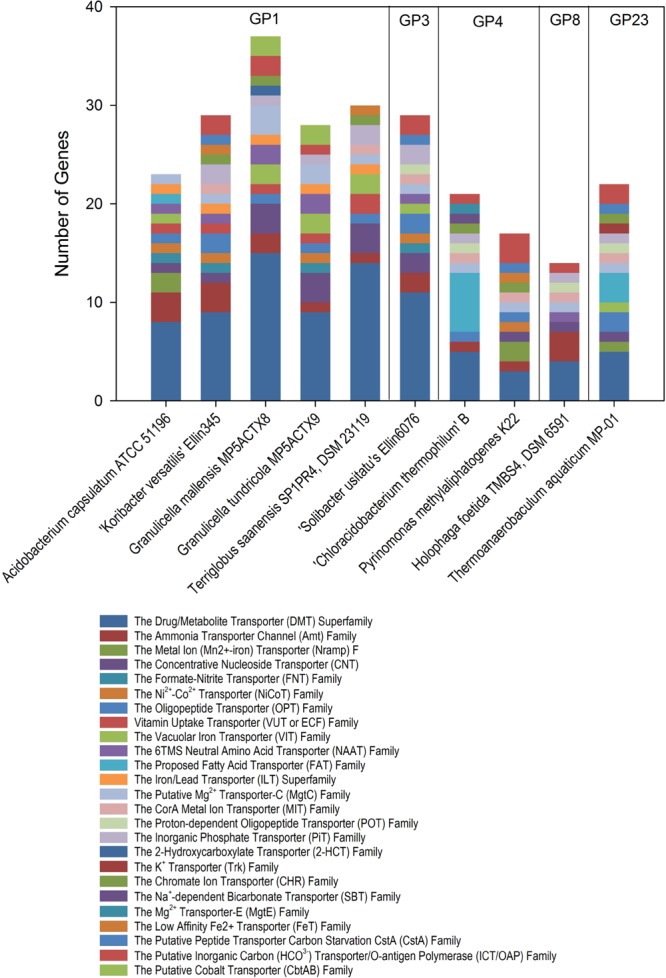
**Families of transporters (TC:3.A) in different *Acidobacteria* subdivisions (GP) genomes.** The comparisons were done using IMG: the integrated microbial genomes database and comparative analysis system.

*Acidobacteria* have a large proportion of genes encoding for transporters (Challacombe et al., 2011). The comparison of three acidobacterial genomes (two A*cidobacterium capsulatum* subdivision 1 and one Ellin6076 subdivision 3) showed about 6% of the total coding sequences are transporters (Ward et al., 2009). The Carbohydrate transport and metabolism category given by cluster of orthologous groups (COG) classification can range from 8.6% in *Terriglobus saanensis* type strain SP1PR4T (Rawat et al., 2012b) to 9.18 % in *Granulicella mallensis* type strain MP5ACTX8T (Rawat et al., 2013). An overview of transporter families in 10 acidobacterial genomes is provided in **Figure 4**. The majority of transporters found in acidobacterial genomes belong to the Drug/Metabolite transporter superfamily. The high number of different transport systems facilitates the acquisition of a broad range of substrate categories, including amino acids, peptides, siderophores, cation, or anions. The presence of a broad substrate range of transporters for nutrient uptake suggests an advantage of *Acidobacteria* in complex environments and adaptation to oligotrophic conditions, such as nutrient-limited soil conditions (**Figure 4**).

Although iron metabolism and iron transporters were discussed in genome sequence exploration studies, these characteristics have not been unequivocally demonstrated in culture-based studies. The only direct indication is the observation of iron accumulation in *B. elongata* (Dedysh et al., 2012). Based on genome content, we speculate that the representatives of subdivisions 4, 8 and *B. aggregatus* may also be able to uptake ferric iron, and genes encoding transport systems involved in translocation across the outer and cytoplasmic membranes, i.e., cobalamin/Fe^3+^-siderophore uptake transporters and Fe^3+^ hydroxamate transporters, have been identified in these genomes. Genomic analyses also suggest that *Acidobacteria* may release siderophores to scavenge iron from soil minerals by formation of Fe^3+^ complexes that can be taken up by those transporters or use siderophores from other microorganisms.

## Ecological Inferences Derived From Metagenomic Approaches

Large genome fragments recovered by metagenomics may contain intact metabolic pathways for mining ecologically relevant genome fragments from the environment. The first acidobacterial metagenomic insert was described by Liles et al. (2003). From a bacterial artificial chromosome (BAC) library, 12 (out of 24,400) clones were identified as containing acidobacterial 16S rRNA sequences (nine clones from subdivision 6, two from subdivision 4, and one from subdivision 5), and one clone affiliated with subdivision 5 was selected for full sequencing. Up to date there is no representative isolate available for this subdivision 5. The annotation of 20 ORFs revealed genes involved in cell cycling, cell division, folic acid biosynthesis, DNA repair, and an ABC transporter. In addition, a novel 1,4-butanediol diacrylate esterase gene was found with 40% sequence identity to an esterase from *Brevibacterium linens*. This enzyme is known to catalyze the conversion of insoluble butanediol diacrylate to a hydrolyzed soluble form for the use as a carbon source, suggesting that the bacterium containing this fragment may possess this capability.

Another six acidobacterial genomic fragments (four out of six related to subdivision 6) were recovered from a sandy ecosystem (Quaiser et al., 2003). Interestingly, two of the recovered clones affiliated with subdivision 6 contained regions of homology encoding a tyrosyl-tRNA synthetase, a metal-depending protease, as well as eight or nine purine biosynthesis proteins. In a subsequent examination of fosmid libraries from deep sea sediments (Quaiser et al., 2008), recovered this same syntenic region in eight out of 11 acidobacterial genome fragments affiliated with subdivision 6. In a metagenomic study of *Acidobacteria* in a former agricultural soil, an additional four out of 17 fosmids (from a library of 28,800 clones) were recovered with the same genomic region (Kielak et al., 2010). Thus, it appears that a large percentage of the subdivision 6 *Acidobacteria* members present in both terrestrial and marine environments, contain this conserved genomic region adjacent to their rRNA operons. However, the ecological and evolutionary significance of this striking pattern remains unknown.

In a study designed to recover genes encoding the synthesis of *N*-acyl homoserine lactones (NAHL), Riaz et al. (2008) identified a *qlcA* gene with a lactonase activity for the degradation of NAHLs. Sequencing of the genomic fragment containing this gene revealed that nine out of 20 ORFs were related to sequences derived from members of *Acidobacteria*. Similarly, genes involved in polyketide synthesis pathways were identified from a fosmid library in cloned inserts containing genes significantly similar to genes from ‘*S. usitatus*’ (Parsley et al., 2011). Primers targeting the *mta*D homolog (encoding protein involved in myxothiazol biosynthesis) identified in a metagenomic library were also used to screen acidobacterial isolates. In four out of six isolates examined (belonging to subdivisions 3, 4, and 6) *mta*D homologous sequences were identified, suggesting widespread distribution of PKS pathways among *Acidobacteria* (Parsley et al., 2011). Genes involved in PKS biosynthesis were also identified in sequenced acidobacterial genomes (Ward et al., 2009). The only report of antibacterial metabolites in relation to genomic potential was described by Craig et al. (2009), who isolated and characterized metabolites derived from acidobacterial genome fragments hosted by *Ralstonia metallidurans*. These compounds showed inhibitory activity against *E. coli, Bacillus subtilis*, and *Staphylococcus aureus*. However, further analysis revealed that the novel metabolites reported by these authors appeared to arise from a mixed biosynthetic pathway involving both the type III polyketide synthase encoded by acidobacterial genome fragments and endogenous *R. metallidurans* enzymes. Multiple reports on PKS pathways identified in *Acidobacteria* suggest widespread distribution of such pathways across this phylum, suggesting a role in the persistence, resistance and abundance of these bacteria in soil ecosystems. However, without the characterization of the polyketide products, the production of antimicrobials by *Acidobacteria* remains speculative.

A moderately thermostable lipase (optimum temperature between 50 and 60°C) from a member of *Acidobacteria* phylum was also described by metagenomic approach from forest soil (Faoro et al., 2012). Interestingly, phylogenetic analysis revealed that the lipase-encoding gene was of fungal origin and was acquired via horizontal gene transfer. In total, large-insert metagenomics analyses have served to recover numerous acidobacterial genomic regions, but the actual ecological insights offered from such fragmented datasets remain limited.

## Environmental Surveys that Correlate *Acidobacteria* Distribution with Environmental Factors or Constraints

**FIGURE 5:**
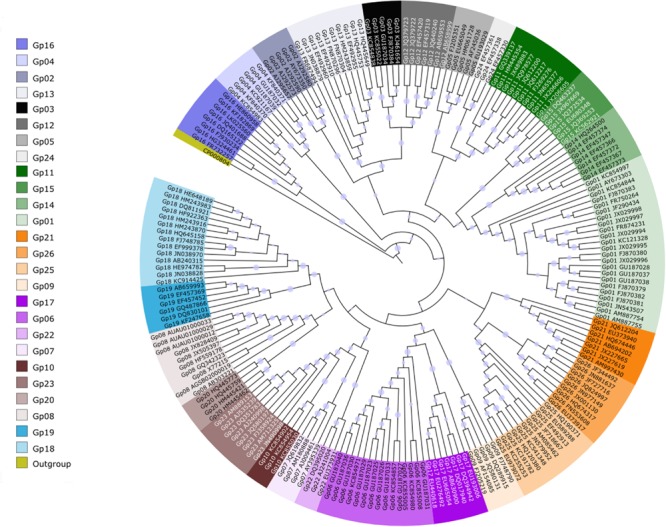
**The *Acidobacteria* subdivisions phylogenetic tree.** The phylogenetic tree is based on 220 sequences of 26 different subdivisions (GPs) of *Acidobacteria* from Silva database (http://www.arb-silva.de/) classified by RDP classifier. The sequences were aligned in Clustal X12 and the selection of conserved blocks from multiple alignment was carried out by Gblocks (Talavera and Castresana, 2007) for phylogenetic analysis. The phylogenetic tree was based on Neighbor-Joining clustering algorithm with 1000 bootstrap. Circles in the branches of the tree represent bootstrap support more than 75%. Outgroup is *Castenholzii roseiflexus*.

Given the high diversity within the *Acidobacteria* phylum, as well as within particular subdivisions (**Figure 5**), it is expected that they also represent a wide range of physiological traits, as observed for other highly abundant and diverse bacterial phyla, such as the *Proteobacteria*. However, most of the studies up to date focus on *Acidobacteria* at phylum level leading to gross generalizations that may not mirror the ecological traits representative of lower taxonomic levels. Nevertheless, some general trends have been discerned from such broad-level analyses. In the most expansive study conducted to date, pyrosequencing of 16S rRNA gene fragment was used to examine the biotic or abiotic factors that most influence the abundance, diversity and composition of soil acidobacterial communities in different types of soils (88 types; Jones et al., 2009). Fierer et al. (2007) studies have shown, based upon 16S rRNA gene sequence distributions across 71 soils differing in geochemical characteristics, that the abundance of *Acidobacteria* was generally higher in soils with very low resource availability (low C mineralization rate) and that the proportions of *Acidobacteria* were higher in C-poor bulk soils in comparison to the rhizosphere. However, this position has been challenged by Jones et al. (2009) and Navarrete et al. (2013b) who found a positive correlation between acidobacterial abundance and organic carbon availability. At the phylum level, many studies have shown that *Acidobacteria* is sensitive to inorganic and organic nutrients inputs (Cederlund et al., 2014; Koyama et al., 2014; Pan et al., 2014; Navarrete et al., 2015) and *Acidobacteria* seemed to have a role in recovering soils as beneficial to soil nutrient cycling and plant growth after drastic disturbance (Huang et al., 2015).

A number of studies have compared acidobacterial distribution and diversity in relation to proximity to plant roots or plant exudates. Numerous studies based on 16S rRNA sequences have shown a higher proportion and diversity of *Acidobacteria* in the bulk soil as compared to in the rhizosphere (Marilley and Aragno, 1999; Sanguin et al., 2006; Fierer et al., 2007; Singh et al., 2007; Kielak et al., 2008). Other studies have shown *Acidobacteria* abundant in red pepper (*Capsicum annuum* L.) rhizosphere (Jung et al., 2015). Additionally, it was shown by means of shotgun metagenomics that *Acidobacteria* is overrepresented in the soybean rhizosphere as compared to the bulk soil (Mendes et al., 2014).

In several cases, *Acidobacteria* have appeared to tolerate various pollutant such as PCBs and petroleum compounds, linear alkylbenzene sulfonate, *p*-nitrophenol, (Abed et al., 2002; Paul et al., 2006; Sanchez-Peinado et al., 2010) and heavy metals (Ellis et al., 2003; Gremion et al., 2003; Barns et al., 2007) leading to speculation that *Acidobacteria* may be involved in the degradation of certain pollutants. However, although the relative abundance of *Acidobacteria* has often been correlated to specific pollutants, no data, up to date have been reported that support actual activities related to pollutant degradation. Such pollutants appear to have little effect on acidobacterial diversity levels. For example, uranium-contaminated soil possessed an extremely broad diversity of acidobacterial populations, and these soils were actually the basis for the expansion of acidobacterial subdivisions to 26 (Barns et al., 2007).

With the broader characterization of subdivisions and increasing depth of coverage it has now become possible to break down analyses to the subdivision level, which can be far more informative. The available studies provide distribution patterns of different acidobacterial subdivisions across different environmental gradients such as pH, nutrients, and carbon.

Especially striking is the predominance of *Acidobacteria* in low pH conditions, in particular members from subdivision 1 (Sait et al., 2006). On the other hand, Barns et al. (1999) suggested that some acidobacterial subdivisions had an aversion to low pH conditions in soil and other case, subdivision 6 can be either positively or negatively correlated with soil pH (Chan et al., 2006; Mukherjee et al., 2014). The abundance of subdivisions 1, 2, 3, 12, 13, 15 was negatively correlated with soil pH, yet subdivisions 4, 6, 7, 10, 11, 11, 16, 17, 18, 22, 25 showed a positive correlation. Similarly, terminal restriction length polymorphism (T-RFLP) and cultivation strategies detected a significant decrease of *Acidobacteria* from subdivisions 1, 2, and 3 at soil pH higher than 5.5 (Männistö et al., 2007). This trend appears also to hold for cold-adapted populations of *Acidobacteria* (Lipson and Schmidt, 2004; Männistö et al., 2007). Similar trends have been found in other environments as well, and *Acidobacteria* from acid mine drainage systems seem to be even better adapted to more acidic conditions (pH 2–3) than *Acidobacteria* from soil environments (Kleinsteuber et al., 2008). Furthermore, it has been observed that the phylogenetic diversity of acidobacterial communities becomes increasingly constrained as soil pH deviates from neutrality (Jones and Martin, 2006). A possible explanation may lie in increased cell specialization and enzymes stability at more extreme pH, where closely related *Acidobacteria* may share similar cellular strategies to deal with discrepancies between intra- and extra-cellular pH. Despite this strong correlation with pH, it is not yet clear if this represents a direct causal relationship or if it is the result of other environmental factors that co-vary with pH.

In fact, by measuring soil factors such as Al, Ca, Mg, K, B, and micronutrients that had not been taken into account in previous studies, Navarrete et al. (2013b) demonstrated that subdivisions 4, 6, and 7 may actually respond to decreases in soil aluminum and soil Ca and Mg in tropical soils. The subdivisions 6 and 7 responded to high contents of soil Ca, Mg, Mn, and B, what elements are required for the growth of all living organisms. Magnesium ions are required by large number of enzymes for their catalytic action, including all enzymes utilizing or synthesizing ATP, or those that use other nucleotides to synthesize DNA and RNA. However, the ionic magnesium cannot directly be up taken by the biological membranes because they are impermeable to magnesium (and other ions), so transport proteins must facilitate the flow of magnesium and other ions, both into and out of cells (Beyenbach, 1990). Future studies on *Acidobacteria* subgroups 4, 6, and 7 functions certainly will elucidate the role of those subgroups in soil. In addition, subdivision 10 has been shown to correlate with soil factors linked to soil acidity such as pH, Al, and Al saturation while subdivision 13 was correlated with soil P, B, and Zn (Navarrete et al., 2013b). The subdivision 2 has been reported in Amazonian forest soil (Navarrete et al., 2013b) and in Mata Atlantica and Cerrado soils (Catao et al., 2014) correlated with Al^3+^ levels and CO_2_ concentration. Aluminum in tropical and subtropical soils is toxic to crops (Yachi and Loreau, 1999). This might suggest that subdivisions 2 and 10 may have metabolic tolerance to aluminum in soil. Subdivision 4 abundance tends to increase with increasing soil pH, suggesting different physiologies for members of this subdivision (Foesel et al., 2013). Thus, it is important to treat soil pH as is a master variable that is related to additional changes in other soil factors, such as Al concentration and macro- and micro-nutrient availabilities (McBride, 1994), which may represent the actual drivers for observed microbial community dynamics.

Navarrete et al. (2013b) and Pessoa-Filho et al. (2015) showed that different subdivisions showed disparate correlations with respect to soil nutrient or chemical status. Although subdivision 1 has negative correlations with P, C, and N, members of subdivisions 5, 6, and 17 appeared to be high abundant in more nutrient-rich soils. Similarly, Männistö et al. (2012) observed phenotype dependent responses (within members of subdivisions 1 and 2) to seasonal changes in Arctic tundra soil ecosystem, which were related to nutrient and carbon availability. Foesel et al. (2013) provided culture-independent evidence for a distinct niche specialization of different *Acidobacteria*, even from the same subdivision, due to particular soil physicochemical (pH, temperature, nitrogen or phosphorus) and biological parameters (respiration rate, abundances of ciliates or amoeba, vascular plant diversity) in grassland and forest soils. Pessoa-Filho et al. (2015) showed the natural dominance of acidobacterial subdivisions 4 and 6 correlated with Mg and Ni contents present in serpentine tropical savanna soil. Additionally, da Rocha et al. (2013) showed that the *Holophagae* (subdivision 8) respond to leek roots (qPCR based study) either by increased cell division and/or by increase in genome quantity per cell.

## Are Acidobacteria Oligotrophs?

The strong negative correlation between the abundance of *Acidobacteria* and concentration of organic carbon in soil has led to the conclusion that members of this phylum may be oligotrophic bacteria. However, it was pointed out that not necessarily all members would be oligotrophic (Fierer et al., 2007). In addition, genome sequences revealed the presence of only one or two copies of 16S rRNA genes suggesting lower growth rates, which has been previously correlated with oligotrophy (Klappenbach et al., 2000; Stevenson and Schmidt, 2004; Eichorst et al., 2007; Kleinsteuber et al., 2008; Ward et al., 2009). It is important to mention that although cultivation strategies regarding *Acidobacteria* isolates are often referring to nutrient limiting media (Janssen et al., 2002; Stevenson et al., 2004; Davis et al., 2005) some of the isolates were shown to be able to grow in higher carbon sources concentrations (Eichorst et al., 2007; de Castro et al., 2013).

The two observations mentioned above (negative correlation of *Acidobacteria* with organic carbon and lower growth rates) are also consistent with the ecological role of K-strategists. It has been predicted that K-strategists would prosper in environments with low abundance of nutrients, which is not the same as to say that they are oligotrophs (Andrews and Harris, 1986). Compared to r-strategists, K-strategists are predicted to have lower growth rates, but high efficiency in converting nutrients to biomass as well as high tolerance to toxic compounds, among other characteristics. Microbes that present the ecological K strategy are better competitors in oligotrophic environments (Andrews and Harris, 1986). Although the term oligotroph has been used in different ways, it usually describes an organism that is not able to grow or thrive in environments with high nutrient concentrations (Koch et al., 2008). However, it may be premature to assume that all *Acidobacteria* have the same ecological strategy, since metagenomic data and physiological description of different subgroups have indicated that there is a high variation within this phylum.

## Acidobacterial Interaction with Other Microbes

Additional evidence for interaction with soil bacteria is the fact that *Edaphobacter aggregans* and *B. elongata* were isolated from co-cultures with methanotrophic bacteria. It was demonstrated that *B. elongata* was unable to use CO_2_ and other C1 carbon compounds, which would be produced by the methanotrophic partner. Instead, it was proposed that the *Acidobacteria* was using the exopolysaccharides produced by the methanotroph as carbon source (Koch et al., 2008; Dedysh et al., 2012).

It is suggested that there is ecological relationship between *Acidobacteria* and *Proteobacteria* because they are often observed to be intimately associated with each other in the environment, and may influence each other’s position in the community. Meisinger et al. (2007) observed, via Fluorescence *in situ* hybridization (FISH) counts, that members of subdivisions 7 and 8 were always associated with epsilon or gamma-proteobacteria in filamentous microbial mats in hydrogen sulfide-containing springs. It was therefore hypothesized that the *Acidobacteria* often live as chemo-organotrophs in association with the autotrophically fixed carbon in the poorly oxygenated regions. Enrichment strategies have also often recovered consortia comprised of *Acidobacteria* and *Proteobacteria*, as exemplified by the co-cultivation of subdivision 6 members from freshwater lake sediments with *Alphaproteobacteria* (Spring et al., 2000). However, it is not yet clear if co-cultivation stems from overlapping niches between the different consortium members or if they have necessary metabolic interactions. It has been suggested that co-cultures containing acidobacteria should be studied more closely to reveal potential ecological interactions and growth preferences (Stevenson et al., 2004). Also, given advances in sequencing, such enrichment cultures should be able to yield full genome sequences of a much broader range of acidobacteria that available in pure culture. Certain groups of *Proteobacteria* have been associated with copiotrophic lifestyles and given this association, Smit et al. (2001) hypothesized that the ratio between *Proteobacteria* and *Acidobacteria* (P/A) may provide insight into the general nutrient status of soils. Low P/A ratios would be indicative of oligotrophic soils, while high ratios would be observed under copiotrophic conditions.

## Conclusion and Future Directions

The high abundance and ubiquity of *Acidobacteria* in soils raises questions related to the physiological traits that have led to this marked success. Although genome sequences have provided important information, our integration of genomes has often not been informed by studies, and genomics analyses remain highly skewed *Acidobacteria* subdivision 1, the groups for which most cultures are available. There is therefore an urgent need to isolate and sequence genomes of representatives from other subdivisions in order to understand their basic characteristics. Due to the still problematic cultivation of *Acidobacteria*, techniques like micro-cultivation and single-cell sequencing should give steps forward to obtain a more representative range of acidobacterial genomes. In addition, with the increased high throughput of shotgun metagenomic studies and associated postgenomic, it should be possible to start dissecting acidobacterial genomes from environmental datasets, thereby circumventing the necessity for cultivation. As an intermediate step, metagenomic analyses of more simplified systems, such as enrichment and non-axenic cultures should also yield access to important genome information. It must, however, be stressed that cultivation efforts remain a top priority, as these provide the necessary material for physiological studies and confirmations of genomic predictions. The 16S rRNA data provided by next generation sequencing together with soil chemicals (macro and micronutrients) can help to elaborate specific culture medium for different *Acidobacteria* subdivisions isolation. Despite the limitation of current genome-based studies, the genomes obtained to date still give important hints related to the factors that explain the successful adaptation of this phylum to harsh soil conditions. These factors include the large number of high-affinity transporters, the potential utilization of a wide variety of carbohydrates as substrate, the resistance to antibiotics and production of secondary metabolites, the production of EPS and the potential use of bacterial produced polymers such as gellan gum.

Although little direct evidence, genomic studies reveal that decomposition and utilization of natural polymers such as chitin, cellulose, EPS, and gellan gum as potential important aspects for future studies. Also better knowledge about the production of EPS, biofilm, and secondary metabolites in *Acidobacteria* subdivisions is of importance to understand the survival, resistance, persistence in soil as well as possible interactions of members of this phylum with other soil microorganisms. As *Acidobacteria* are ubiquitous, they should interact, positively or negatively, with other soil habitants. Therefore, unraveling these interactions is vital for the proper understanding of their role in terrestrial ecosystem functioning.

Additionally, the recovery of 16S rRNA genes from the environment should be taken forward. These surveys have provided new insight in terms of distribution of different acidobacterial subdivisions and relation to environmental variables, but more experimental approaches need to be coupled with high throughput toolbox to tease out the actual roles of environmental variables. Further, more molecular studies that attempt to look at activities such as metatranscriptomic and stable isotope probing (SIP) approaches might be considered.

## Author Contributions

Analyzed the data: AK, CB, and EK. Contributed reagents/materials/analysis tools: JvV and EK. Wrote the paper: AK, CB, GK, JvV, and EK.

## Conflict of Interest Statement

The authors declare that the research was conducted in the absence of any commercial or financial relationships that could be construed as a potential conflict of interest.
