# Supplementary material for: The Ecology of Acidobacteria: Moving beyond Genes and Genomes
Source: Front Microbiol. 2016 May 31;7:744. doi: 10.3389/fmicb.2016.00744 (PMC4885859; doi:10.3389/fmicb.2016.00744)
Supplement: Supplementary file 1 [file Table_1.PDF]

Table S1 Enzymatic profile of *Acidobacteria* determined by API ZYM detection of enzymes system

GP	Genus	α-Galactosidase	β-Galactosidase	β-Glucuronidase	α-Glucosidase	β-Glucosidase	N-Acetyl-b-glucosaminidase	α-Mannosidase	α-Fucosidase
	Acidobacterium	+	+	+	+	+	+	-	-
	'Acidipila'	+	+	-	+	+	+	-	+
	Acidicapsa	+	+	+	+	+	+	+	+
1	*Edaphobacter*	+	+	+	+ 1	+1	+ 1	-	+
	Terriglobus	+	+	+	+	+	+	+6	+
	Granulicella	+2	+	+	+3	+	+3	+4	+4
	Bryocella	ND	+	+	+	+	+	ND	ND
	Telmatobacter	+	+	+	ND	+	+	-	+
	'Occallatibacter'	+	(+)	+	+	-7	-7	+	+
	'Silvibacterium'	ND	+	+	+	+	+	ND	ND
	'Terracidiphilus'	ND	+	+	+	+	+	ND	ND
3	*Bryobacter*	-	+	-	-	+	+	+	+
	Paludibaculum	-	+	-	-	+	+	-	+
	Pyrinomonas	-	-	+	+	-	+	-	-
4	*'Blastocatella'*	-	-	(+)	-	-	(+)	-	-
	Aridibacter	-	-	(+)[5]	(+)	+	+	-	-

Acidobacteria from subgrousp 8, 10, and 23 were not tested with API ZYM (Biomeriux). [1] negative or weak for *E. aggregans* and *E. dinghuensis.* [2] negative for *G. rosea and aggregans.*[3] negative for *G rosea* .[4] negative for *G maleensis.*[5] negative for *A. kavangonensis.* [6] *Negative para T. albidus.* [7] Negative for *O. savannae.* Detailed results in supplemental material
